# Enabling evidence to tackle everyday diseases to mitigate another pandemic

**DOI:** 10.12688/gatesopenres.13497.1

**Published:** 2022-04-07

**Authors:** Trudie Lang

**Affiliations:** 1 Centre for Tropical Medicine and Global Health, Nuffield Department of Medicine, University of Oxford, Oxford, OX3 7FZ, UK

**Keywords:** pandemic preparedness; health research; outbreaks; global research equity, health research capacity; capacity strengthening

## Abstract

The next emergent novel pathogen is likely to occur where the ability to undertake health research and collect life-saving data is lacking. Without embedded and ongoing research activities in place spotting and stopping a new threat is not possible, thereby enabling undetected infection and unchecked transmission within a community. Without local existing capabilities to collect such data delay is catastrophic. Fundamental goals in pandemic preparedness should be to stop an outbreak before it becomes a pandemic. This requires immediate action from teams already in place with the right skills, this could be readily achieved if we shift our thinking and enable research capabilities to be present in every healthcare setting. Addressing fundamental gaps in health research capacity and equity could tackle this and then we would be better prepared, globally.

## Disclaimer

The views expressed in this article are those of the author(s). Publication in Gates Open Research does not imply endorsement by the Gates Foundation.

## We need a whole ecosystem of health research studies

The need for the whole ecosystem of health research studies is true for any disease, and in an outbreak, we need these data all at once. During the coronavirus disease 2019 (COVID-19) pandemic there was fast and strong investment in hospital based clinical trials that evaluated therapies. These were highly successful for improving outcomes in severe disease ^1, 2^ but failed to generate effective, accessible and affordable anti-viral therapies that could be given before the disease progressed ^1, 2^. Anti-viral therapies typically work by stopping replication and therefore are ideally given very early in the infection. It is important to collect all types of health research data, yet funding was highly siloed geographically and in the questions being addressed ^3, 4^. The most important element in preventing a pandemic is to first be able to identify a new threat and then as quickly as possible characterise how the disease presents and how transmission can be blocked. These steps need surveillance in place and the capability to undertake observational studies. Addressing gaps in ability and ensuring funding is directed to the full range of health research are essential to mitigate another pandemic.

**Figure 1.**
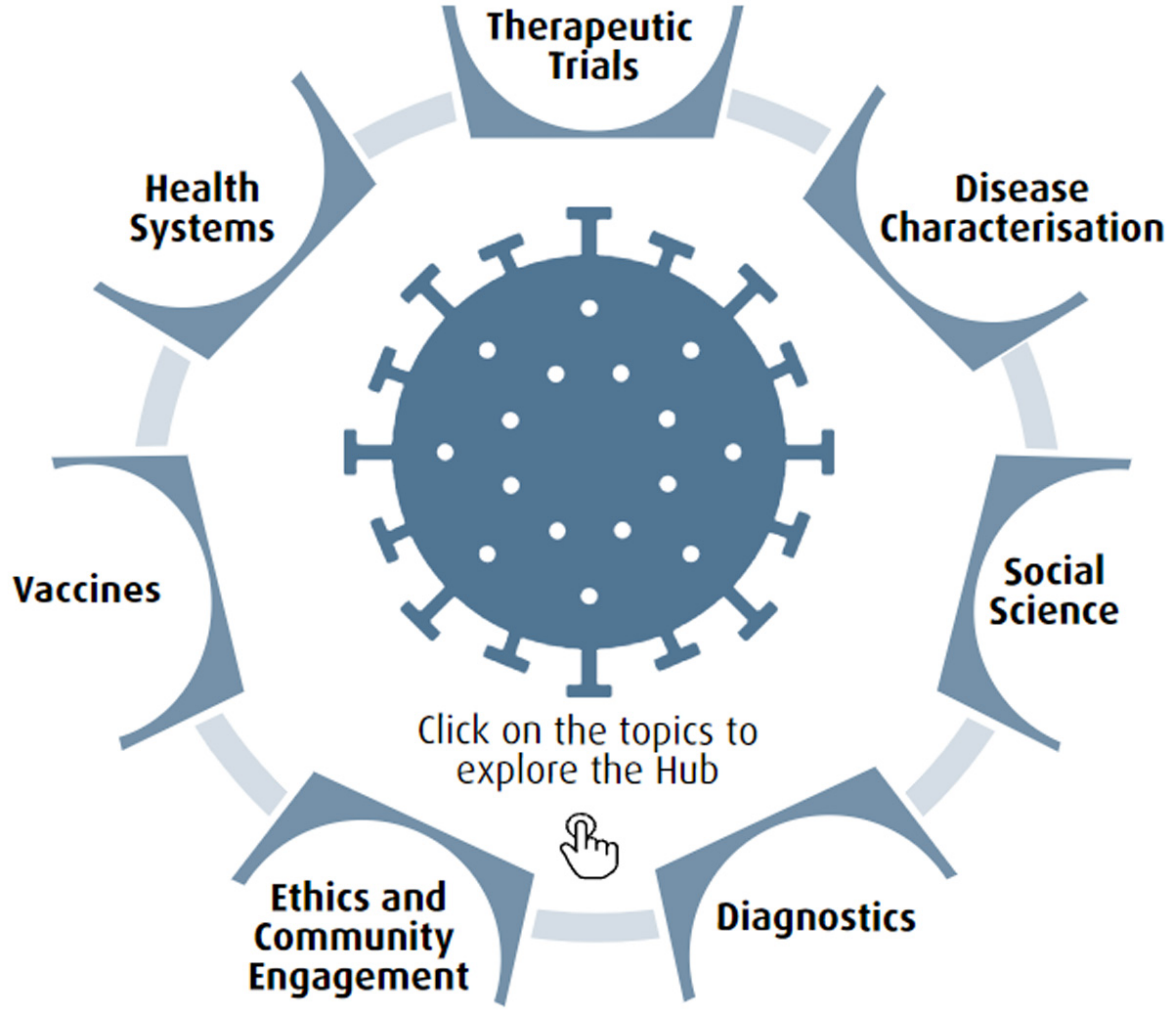
The COVID Research Implementation Hub: Infographic used to direct research team to protocols, tools, training and guidance during the coronavirus disease 2019 (COVID-19) pandemic across all the different types of studies that were needed.

The skills, protocols, methods and resources for all health research studies are readily adaptable between diseases as has been reported and put into practice ^5^. The International Severe Acute Respiratory and emerging Infection Consortium (ISARIC) developed a disease characterisation protocol for use firstly in severe acute respiratory syndrome (SARS) ^6^. This was then adapted for the Ebola outbreak, this was then again pivoted for Zika and taken up successfully across the globe for COVID-19 ^7– 9^. The Zika outbreak highlights the importance of all forms of health research data. In this situation there were in fact no clinical trials as there were no drugs or vaccines to evaluate. Here at the outset, we initially faced an unknown pathogen causing devastating microcephaly in babies, the cause needed to be determined and then the nature of transmission and the harm that was being caused needed to be fully understood. The studies undertaken through the Zika outbreak that answered these questions included public health and social science studies, vector biology, genomics and disease characterisation. This shows us how all types of health-related research data should be valued equally and that clinical trials are just one element of the health research ecosystem, that needs to be considered as a whole, comprised of these critical elements. Figure 1 is an infographic used on the COVID Research Implementation Hub to direct research teams to protocols, tools, training and guidance during the COVID-19 across all the different types of studies that were needed.

We can consider this connected ecosystem further because once we have interventions, such as drug and vaccines we need to rapidly undertake well designed clinical trials that will tell us if these new interventions work and are safe. For a successful trial that produces strong evidence on the safety and efficacy of an intervention in any given community, then we need data on the nature of the disease and the epidemiology in different specific settings to guide the design of good protocols. Alongside these it is essential to firstly and then in parallel run social science-based studies to understand community perceptions, and to develop and test diagnostics, understand the pathogen and prevent transmission, etc. Therefore, a connected ecosystem of studies are required to generate the full range of data that we need. Clinical trials are key at the end of the process but they do not occur in isolation and other data sets are just as important. This point is especially relevant to consider in low-resource settings where the ability to run trials must be considered alongside the ability to collect all the other types of data needed to design the right trials in the right place if we are to develop drugs and vaccines that will make a difference in varied patient populations.

## The skills and systems needed are the same for any disease and so wider benefit and impact would be achieved

We know from our research and long-standing experience in addressing these gaps that the barriers to undertaking health research are the same irrespective of disease, region, organisation or type of study ^3, 4^. This is good news because if we put in place highly practical solutions to tackle everyday disease priorities and burdens, then they are active and will spot a new threat. These teams can then pivot from their current research to focus on the new pathogen and stop it in its tracks before it escapes.

Many organisations, including the World Health Organisation, research consortia and funders, such as the European and Developing Countries Clinical Trial Platform (EDCTP) and the Bill & Melinda Gates Foundation (BMGF), are working with frontline health workers to deliver the ability to undertake research within their care delivery practices ^10– 12^. Pragmatic research studies can be undertaken in any setting by all roles and levels of staff to measure the scale and burden of diseases that impact their communities, or identify and test practical and locally appropriate new treatments, care plans, or prevention approaches to address them. This approach is being delivered across Africa, Latin America and Asia and has resulted in new evidence for diseases such as tuberculosis (TB), malaria and dengue fever. These accessible and highly applicable initiatives drive changes such as better community-based practices to reduce maternal and new-born fatalities. With practical research skills and awareness in place, teams, such as the REDE: The Latin America and Caribbean Network for Research Capacity Strengthening, have already pivoted their skills in-situ to work on Ebola, Zika and more recently COVID-19 showing that this approach works. Having research capabilities actively working within healthcare system is the most effective mechanism for a new outbreak to be spotted and the data captured immediately to determine how to stop further spread and manage the resultant cases. These same teams will also be ready with the skills and systems in place to run trial when products come through, which can be designed appropriately as there is local epidemiology and social science data.

Conversely, if these easily taught skills are absent the delay in detection and response could be the difference between an outbreak and a pandemic.

## Thinking wider than pandemics will prevent a pandemic

Teaching skills to community health workers to survey, detect and assess an emergent new threat and putting response systems and protocols in place that sit in wait for a new event will not work. These skills need to be in practice, be present and embedded into healthcare delivery, rather than considered different and distinct from daily work and put away for the moment a response is needed.

Thankfully, these skills are no different than are needed for tackling ongoing, daily diseases that impact communities. Also, teaching and implementing these is neither difficult or expensive and can be undertaken at scale if we connect and build on the success of existing research networks and knowledge mobilising initiatives. We can develop this further by extending, connecting or setting up, new networks where there are gaps, which are then able to use and adapt infrastructure, expertise and extensive reach and reputation in low- and middle-income countries (LMICs) bringing a ready-made and proven cross-function resource for knowledge sharing, training and building communities of practice across and between partners, topic areas and research methods. This can bring immediate impact through tackling ongoing disease burdens, and then ready to immediately act in the event of a new threat.

## Achieving global research equity

Lastly, COVID-19 at least highlighted to everyone the importance of health research. Whilst there has been incredible mobilisation to understand and mitigate COVID-19, this has also highlighted the ongoing stark and profound gaps. It remains true that 90% of all health research benefits only 10% of the world’s population, primarily those in the wealthy north ^13^. Diseases of poverty lack crucial evidence to change the appalling outcomes these illnesses bring to the poorest communities across the globe. It is readily achievable to work within healthcare teams in these settings to implement locally-led research in order to understand and develop interventions to tackle the diseases that devastate families and economies in these woefully underserved regions of the world. There is much that can be readily done to enable research teams in the global south to not get left behind and for research to happen at the same time everywhere. We can change how research is funded and rewarded to recognise team science and foster truly federated global partnerships. We can also work to ensure that funding flows across a required matrix to tackle every unanswered question and is not siloed geographically or in funding similar trials in the same settings. Low-income countries should have equitable access to the benefits of research and in taking part. Furthermore, unless research capacity is in place then the global risk is greater and we are not globally prepared.

## Data availability

No data are associated with this article.
