# Supplementary material for: Enabling evidence to tackle everyday diseases to mitigate another pandemic
Source: Gates Open Res. 2022 Apr 7;6:45. [Version 1] doi: 10.12688/gatesopenres.13497.1 (PMC9296833; doi:10.12688/gatesopenres.13497.1)
Supplement: Peer review history

# Peer review: Reviewer response for version 1

**DOI:** 10.21956/gatesopenres.14760.r32136

**Published:** 2022-07-19

**Authors:** Palmer Masumbe Netongo, Wilfred Mbacham

**Affiliations:** 1 Molecular Diagnostic Research Group, Biotechnology Centre, University of Yaoundé I, Yaoundé, Cameroon; 2 University of Yaounde I, Yaoundé, Cameroon

Reviewers’ comments on the open letter:

1. Well written open letter overall, addressing current key issues of research participation and funding worldwide.
2. Some suggested key points to be addressed:

Under the subheading: “We need a whole ecosystem of health research studies “, in addition to addressing gaps in ability to tackle outbreak and funding, it is very important for the global research community to:

1. Consider allocating funding following geographical repartition of research gaps where there is an urgent need of data worldwide (mainly in the global south, as many programs do works on collecting any type of data which could be beneficial for preparedness of future pandemics: cholera, Marburg, Ebola…).
2. Allocate more research grants to citizens from the global south (institutions, …), as nowadays still many research grants have restrictive selection criteria (be a citizen of the global north…).

Under the subheading: “Thinking wider than pandemics will prevent a pandemic”, the author could emphasize more on the importance of having research capabilities actively working within healthcare systems in the global south (TB, Malaria…) by giving some examples: like making global south countries (communicable or non-communicable disease programs) accountable for their management budget through publications in peer-reviewed journals of implementation activities' results. This might greatly contribute to pandemic preparedness.

Does the article adequately reference differing views and opinions?

Yes

Are all factual statements correct, and are statements and arguments made adequately supported by citations?

Yes

Is the Open Letter written in accessible language?

Yes

Where applicable, are recommendations and next steps explained clearly for others to follow?

Yes

Is the rationale for the Open Letter provided in sufficient detail?

Yes

Reviewer Expertise:

Infectious disease, molecular diagnostics, public health, capacity building.

We confirm that we have read this submission and believe that we have an appropriate level of expertise to confirm that it is of an acceptable scientific standard.

# Peer review: Reviewer response for version 1

**DOI:** 10.21956/gatesopenres.14760.r31923

**Published:** 2022-04-21

**Authors:** Mahesh K. Maskey

**Affiliations:** 1 Nepal Public Health Foundation, Kathmandu, Nepal

Prof. Lang’s central argument in this article is that the generation of evidence is not the prerogative solely of clinical trial. Crucial though clinical trial is, the whole ecosystem of health research needs to be in action for the development our understanding of any disease, particularly if we aim to be prepared for the pandemics of the future. Prof. Lang points to the disproportionate investment of health-research funding in clinical trials during the Covid-19 pandemic, and argues that while the result of this misplaced focus was a success in treating severe cases of the disease, but it also led to less attention to develop anti-viral therapies essential for stopping replication and disease progression. She draws on experience of the responses to the SARS, Ebola, and Zika viruses to underline the importance of surveillance and observational studies in evidence-generation. “The most important element in preventing a pandemic is to first be able to identify a new threat and then as quickly as possible characterize how the disease presents and how transmission can be blocked,” Prof. Lang writes. The data needed, therefore, includes not only the “nature of the disease and the epidemiology in different specific settings,” but information from social-scientific studies of community understandings of disease and transmission as well. Many different types of studies are needed to generate the full range of data, with clinical trials being the endpoint of this process. 

 The priorities of the world’s health-research institutions are increasingly becoming reductionist. Coming from a globally acclaimed expert in clinical trial, this article’s emphasis on a more holistic approach to the generation of evidence is refreshing, welcome, and important. The arguments here appear to be a further development of the ones in Prof. Lang’s July 2020 article in Nature, which drew on a survey of approximately 4000 researchers from 130 countries. In that article, Prof. Lang emphasized the stark geographical inequity of health-research funding investment, with 90% of such research benefitting only 10% of the world’s population. The remaining 90% of the world’s population represent countries where appropriate health research is sorely needed to prevent and manage the devastating effects of pandemics. This is where research capacity needs to be strengthened, and in the article reviewed here Prof. Lang provides examples from different parts of the world to show how that strengthening is possible and within reach. Basic skills in survey, detection, and assessment are easily and inexpensively taught, and are also what is needed for the routine treatment of diseases that impact communities. If these skills are not developed, she warns, then in the years to come, “delay in detection and response could be the difference between an outbreak and a pandemic.” Unless low-income countries have equitable access to research funding and thereby to the benefits of research, and unless research capacity is in place in these countries through a federated global partnership, Prof. Lang advises that the global risk is greater for both the occurrence and management of future pandemics. 

 This important article would be further strengthened by a brief exploration of differing points of view. What are the viewpoints that maintain the current inequity in research-fund allocation, in spite of the advocacy of powerful health institutions such as the World Health Organization? What arguments are made to limit funding allocation to low-income countries? Is one such argument the belief that good research cannot be done in the Global South? Such thinking surely perpetuates a vicious cycle of low research capacity and low research funding. Similarly, what are the viewpoints and mindsets that maintain clinical trial as a grossly over-prioritized arena of health research? Why is there a blind spot with regards to the importance of effective intervention strategies in prevention and in the very early stages of infection when the virus replicates? Why is there such under-prioritization of socio-ethnographic studies and community engagement, despite the evidence that data from these is of critical importance to our ability to serve our communities?

 I believe attention to these queries would have made a more balanced presentation of arguments while strengthening the core message of the open letter by Prof. Lang.

Does the article adequately reference differing views and opinions?

Partly

Are all factual statements correct, and are statements and arguments made adequately supported by citations?

Yes

Is the Open Letter written in accessible language?

Yes

Where applicable, are recommendations and next steps explained clearly for others to follow?

Yes

Is the rationale for the Open Letter provided in sufficient detail?

Yes

Reviewer Expertise:

, Maternal and Child health, Health systems research, Implementation research and Public Health policy.

I confirm that I have read this submission and believe that I have an appropriate level of expertise to confirm that it is of an acceptable scientific standard.
